# Cardio-metabolic disease risk factors among South Asian labour migrants to the Middle East: a scoping review and policy analysis

**DOI:** 10.1186/s12992-019-0468-8

**Published:** 2019-05-02

**Authors:** Shiva Raj Mishra, Saruna Ghimire, Chandni Joshi, Bishal Gyawali, Archana Shrestha, Dinesh Neupane, Sudesh Raj Sharma, Yashashwi Pokharel, Salim S. Virani

**Affiliations:** 1 Nepal Development Society, Bharatpur-10, Chitwan, Nepal; 2 University of Nevada Las Vegas, Las Vegas, NV USA; 3 Tufts University, Medford, MA USA; 4 Department of Public Health, Aarhus University, Aarhus, Denmark; 5 Harvard T Chan School of Public Health, Harvard University, Boston, MA USA; 6 Welch Center for Prevention, Epidemiology and Clinical Research, Johns Hopkins Bloomberg School of Public Health, Baltimore, USA; 7 Institute of Food, Nutrition and Human Health, Massey University, Wellington, New Zealand; 8 Saint Luke’s Mild America Heart Institute, University of Missouri Kansas City, Kansas City, MO USA; 9 Health Foundation Nepal, Lalitpur, Nepal; 10 America Nepal Medical Foundation, Westfield, MA USA; 11 Section of Cardiology, Michael E. DeBakey Veterans Affairs Medical Center, Houston, TX USA; 12 Section of Cardiovascular Research, Department of Medicine, Baylor College of Medicine, Houston, TX USA

**Keywords:** Non-communicable diseases, Diabetes, Cardiovascular diseases, Migrants, Labour, Migration, South Asia

## Abstract

This paper aims to explore the burgeoning burden of cardiovascular and metabolic disease (CMD) risk factors among South Asian labor migrants to the Middle East. We conducted a qualitative synthesis of literature using PubMed/Medline and grey literature searches, supplemented by a policy review of policies from the South Asian countries. We found a high burden of cardio-metabolic risk factors among the migrants as well as among the populations in the home and the host countries. For example, two studies reported the prevalence of diabetes mellitus (DM) ranging between 9 and 17% among South Asian migrants. Overweight and obesity were highly prevalent amongst South Asian male migrants; prevalence ranged from 30 to 66% (overweight) and 17–80% (obesity) respectively. The home country population had a significant CMD risk factor burden. Nearly 14 to 40% have three or more risk factors: such as hypertension (17 to 37%), diabetes (3 to 7%), overweight (18 to 41%), and obesity (2 to 15%). The host country also exhibited similar burden of risk factors: hypertension (13 to 38%), diabetes (8 to 17%), overweight (33 to 77%) and obesity (35 to 41%). Only Nepal, Bangladesh and Sri Lanka have some provisions related to screening of CMDs before labor migration. Further, analysis of policy papers showed that none of the reviewed documents had requirements for screening of any specific CMDs, but chronic diseases were used generically, failing to specify specific screening target. Given the high burden of risk factors, migrants’ health should become an urgent priority. The lack of specific focus on screening during different stages of labor migration should receive attention. The International Labour Organization and the International Office for Migration, through their country coordination teams should engage local stakeholders to create policies and plans to address this concern. Similarly, there is a need for the host country to become an equal partner in these efforts, as migrant’s better cardiometabolic health is in the benefit of both host and home countries.

**Electronic supplementary material:**

The online version of this article (10.1186/s12992-019-0468-8) contains supplementary material, which is available to authorized users.

## Summary

- Migrants are currently targeted for prevention of tuberculosis and sexually transmitted infections; however, new and emerging diseases such as cardiometabolic diseases and their risk factors have largely been overlooked.
- There is very little evidence on the burden of cardiometabolic diseases risk factors among migrants.
- Our paper demonstrates a high burden of cardiometabolic disease risk factors among the labour migrants, coinciding with a similar burden of risk factors in home and host country populations.
- Despite the high burden, they are not prioritized/targeted in the home and host country for their prevention and management. Only a few countries have prioritized services for migrants, and none specifically mentioned any targets for screening.
- Countries, both at the sending and receiving ends of labour migration, should step up to provide comprehensive services to address the cardiometabolic diseases and risk factors.
- The International Labour Organization and the International Office for Migration should engage the Ministry of Health, Labour and Foreign Affairs in both sending and receiving ends to create platforms, policies, and environment that protect migrants’ cardio-metabolic health.

## Background

The Middle East, a region with countries centred on Western Asia and Northern Africa, hosted 32 million migrants in 2015, which is about 80% of their population [1]. South Asia contributes a significant proportion of this workforce [2]. Historically, South Asian migrants’ health has been neglected by both the host and home governments [3]. Only sexually transmitted infections (STIs) and Human Immunodeficiency Virus (HIV) is routinely screened pre-departure [3], because of the threat to populations in the host nations [4]. Other emerging global health threats including non-communicable diseases (NCDs) are ignored [3].

The migrant population usually adopts a sedentary lifestyle, including the use of car instead of walk for commuting, increase in screen time, jobs that require more sitting, and unhealthy eating habits [5, 6]. Migrants in all occupational categories have unfavorable changes in risk factor profiles leading to hypertension (HTN), diabetes mellitus (DM), and obesity.

A review from Western Europe reported that CVD and its determinants, including diabetes and obesity, were highly prevalent among Turkish and Moroccan labour migrant [5]. Another review among global immigrant populations also reported worse cardiovascular health compared to the general population [7]. A 2015 review on the health status of South Asian migrants in the Middle Eastern countries [3] showed a higher burden of NCD risk factors among the migrants as well as on the source population of sending countries. The health and workplace situation of Sri Lankan migrant domestic workers in the Middle East [8], highlighted the limitations of the insurance policy including lack of support on migrant health; and low coverage, length and benefit in host countries [8]. Both reports highlighted low priority of governments to migrants’ health, particularly in relation to NCDs in the Middle East. However, these reports did not discuss an overview of cardio-metabolic disease (CMD), its drivers and future steps to confront. In this paper, CMD refers to CVDs, diabetes, and obesity among other metabolic conditions to avoid repetition on the use of terminologies. A full definition of CMD is available elsewhere [9].

Given that migrant people’s health might be at higher risk of CMD, the programmatic prevention strategies are likely to be different than traditional interventions. Hence, monitoring CMDs trend among the migrant populations can guide effective prevention and management strategies. There is also a need to analyze the existing migration-related policies in the South Asian region. This review assessed the burden of CMDs and their risk factors among South Asian immigrants working in the Middle East.

## Methodology

We conducted a qualitative review using the framework for scoping review by Arksey and O’Malley [10], which was previously used for NCD [11]. The review-process included four phases: i) identifying the research question, ii) identifying potential studies, iii) data abstraction, and iv) collating, charting, summarizing and reporting the results. We developed the framework of the review in consultation with experts (see acknowledgements). A systematic review was not possible because more deliberations are needed in this area. In the initial review of the literature, it is essential to assess the burgeoning CMD risk factors in home and host country and the related policies. Therefore, we came up with four themes, i) CMD risk factors among migrants, ii) CMD risk factors in home countries, iii) CMD in host countries, and iv) migrant health policy in South Asian countries.

**Fig. 1:**
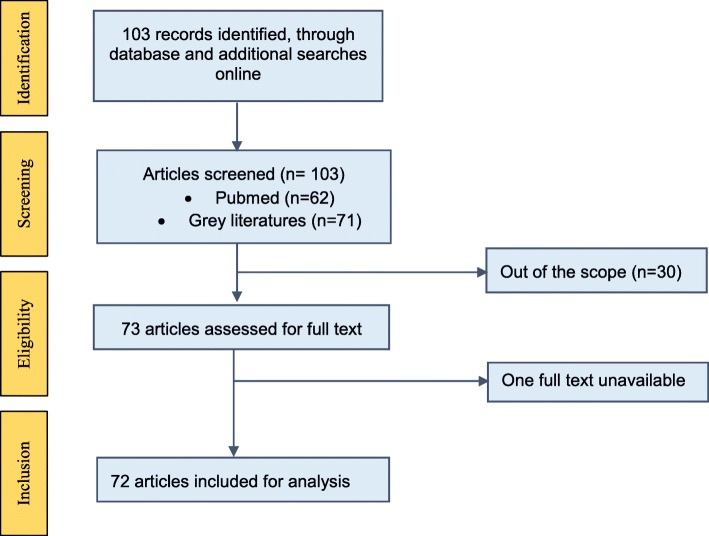
Flow chart of the review process

We searched Medline database up until July 2017 using search terms on (i) migrants’ CMD burden in home and host countries (‘transients and migrants’, ‘cardiovascular diseases’, ‘diabetes’, ‘Middle East’, ‘Asia, Western’); and (ii) CMD burden in the Middle East (‘epidemiology’, ‘prevalence’, ‘cardiovascular diseases’, ‘diabetes’, ‘Middle East’) (See Additional file 1 for search terms used in Pubmed/Medline). We included the grey literature such as World Health Organization (WHO) reports and government publications, searched the WHO websites, libraries, and individual countries websites. Two reviewers selected the articles on mutual consensus (SG, SRM) and abstracted data in an excel sheet (Fig. 1). The inclusion criteria were: must report the burden or the policy on NCD in the home (Nepal, Bangladesh, Pakistan, Bhutan, Maldives, Sri Lanka, Afghanistan, and India) or host countries (Qatar, Saudi Arabia, United Arab Emirates, Jordan, Kuwait, Lebanon, Bahrain). We did not assess the quality of the articles.

We reviewed the migration-related policy from eight countries, with an in-depth policy analysis for Nepal, which is shown in a panel 1. For the panel, we reviewed policy published on the Ministry of Labour and the Ministry of Foreign Affairs website; and from the International Organization for Migration (IOM) and International Labour Organization (ILO). We used a causal loop diagram (CLD)—a tool based on *System Dynamics*-- to further expand on our analysis of CMD epidemiology and migration policies in South Asia [12]. The authors (SRM, SRS) have used CLD in NCD context before [13]. The CLD shows the linkages between social determinants of CMD risk factors, and how these determinants reinforce or balance each other under a prevailing policy context.

### South Asia and migration

South Asia is currently undergoing a rapid demographic and epidemiological transition with a growing burden of NCDs. A total of 13.5% of the region’s population lived below the poverty line in 2015 [14]. The stagnant poverty rates, faltering economic growth and reduced employment opportunities in South Asia have spurred migration to the Middle East for employment. Over the years, migration has played a crucial role in economic development in the region at the micro (household), meso (community) and macro (societal) levels; by providing employment and remittances [1, 2]. South Asia is the second highest remittance receiving region. Remittance is the main source of foreign exchange, larger than any foreign direct investments and foreign aids in this region [15].

India and Pakistan are the two largest labour sending countries in this region [15]. The demographics of the workforce are poorly understood. While Sri Lanka has a high female labour migration, all other countries’ migration is male dominated [8]. A study conducted among 408 Nepalese migrants who worked for six months or more in three Gulf countries: Qatar, Saudi Arabia, United Arab Emirates (UAE), reported that Nepalese migrants in these countries were mostly young men of 26 to 35 years [16]. Most of the migrants were hired in unskilled jobs, such as laborers, scaffolders, and carpenters. Two-third of them had completed only primary level education and were unskilled or semi-skilled laborers [16].

The prevalence of HTN ranged from 13.6 to 47.9% in this region, with the highest prevalence in Nepal [17]. Prevalence of DM in India was 7.3%; [18] and Nepal had the lowest prevalence at 1.0% in rural areas and 8.1% in urban areas [19]. Rapid urbanization and changes in lifestyle has resulted in demographic, dietary and epidemiological transition in recent years [20, 21]. The traditional food culture of unrefined carbohydrates, fibers, and tubers are replaced by high-calorie foods, processed foods, and a higher proportion of meat [22].

### CMD risk factors in migrants

Table 1 shows the four cross-sectional studies [23–26] on the burden of CMD risk factors among migrant workers in the Middle East. Of the four studies, three were among expatriate migrant workers the government visa screening center in the United Arab Emirates (UAE) [23, 24, 26] and one was a community-based study on Indian migrant workers in Gulf countries (UAE, Saudi Arabia, Qatar, Oman, Kuwait, and Bahrain) [25]. Three studies included only male [23–25], and one study included only female migrant workers [26].

**Table 1:** A literature review on the prevalence of cardio-metabolicdiseases in south Asian migrants and host population in the Middle East

Name of the Study	Study Method	Prevalence of Diseases	Risk Factors	Current Understanding	Future Directions	Ref
Hypertension prevalence, awareness, treatment, and control, in male South Asian immigrants in the United Arab Emirates: a cross-sectional study.	A cross-sectional study among 1375 South Asian (Indian, Pakistani and Bangladeshi) adult (≥18 years) male migrant worker at a government visa screening center in the United Arab Emirates (UAE).	The prevalence of hypertension was 30.5%.	In an adjusted analysis, factors associated with participants’ hypertension status were overweight (OR = 1.43; 95% CI 1.01, 2.01); obesity (OR = 2.49; 95% CI: 1.51, 4.10); central obesity (OR = 2.01; 95% CI 1.37, 2.92); family history of hypertension (OR = 1.51; 95% CI 1.05, 2.17); and walking less than 30 min daily (OR = 1.79; 95% CI 1.24, 2.60).	The prevalence of hypertension among young male South Asian immigrants living in the UAE was high. The awareness, treatment, and control of hypertension within this population were very low.	Future initiatives need to consider the sociocultural, religious, ethnic, and educational diversity of this population in the design, development, and implementation of campaigns, interventions, and strategies. Strategies to improve the awareness and control of hypertension among the migrant workers in the UAE is necessary. The public health interventions should target the maintenance of a healthy body size and regular assessment of blood pressure among these populations.	[23]
Association between acculturation, obesity and cardiovascular risk factors among male South Asian migrants in the United Arab Emirates – a cross-sectional study	Cross-sectional study among 1375 South Asian (Indian, Pakistani and Bangladeshi) male migrant worker at a visa health screening center in Abu Dhabi (UAE)	The prevalence of hypertension and diabetes was 30.5 and 9% respectively.	The crude prevalence of overweight, obesity, and central obesity in South Asian immigrants were 35.4, 9.4, and 63.4% respectively. Overall a small proportion of the study participants reported moderate 26.7% and vigorous, 18.2% physical activity. About 62% never had their blood pressure measured. Around 44% of participants with diabetes and 76% of those with hypertension were not aware of their status.	Overweight, central obesity and hypertension were highly prevalent amongst young South Asian male migrants in the UAE. A diminished ‘Healthy Migrant Effect’ with increased years of residency was observed possibly due to greater acculturation and a transition in lifestyle behaviors.	A validated, contextual- and culturally-specific multidimensional instrument to measure acculturation among South Asian migrant populations in the UAE is lacking. Health initiatives targeting the maintenance of a healthy body size, coupled with regular assessments of glucose control and blood pressure are urgently required in this population.	[24]
Is Migration Affecting Prevalence, Awareness, Treatment and Control of Hypertension of Men in Kerala, India?	A community-based cross-sectional study among 191 male Gulf migrants and 193 non-migrant workers aged 25–64 years in the Kerala state of India. Gulf countries in the study included UAE, Saudi Arabia, Qatar, Oman, Kuwait, and Bahrain.	Age adjusted hypertension Prevalence was 57.6% among migrants and 31.7% among non-migrants. In adjusted analysis, migrants were more likely to be hypertensive (OR 3.00, 95% CI 1.83–4.94) than non-migrants.	Awareness (migrants vs. non-migrants: 43.5% vs. 56.9%, *p* = 0.109), treatment (migrants vs. non-migrants: 34% vs. 53%, *p* < 0.05), and control (migrants vs. non-migrants: 12% vs.48%, *p* < 0.001) of hypertension was lower among migrants compared to non-migrants. Most of the NCD risk factors were higher among migrants compared to non-migrants, although they were not associated with higher prevalence of hypertension among them.	Hypertension was highly prevalent among migrants compared to non-migrants. Comparatively fewer migrants than non-migrants had treatment of hypertension or had hypertension under control. Risk factors for hypertension were significantly higher among migrants compared to non-migrants.	The role of stress in the prevalence of hypertension needs to be explored. Efforts should be made to control hypertension prevalence and increase treatment and control of hypertension among migrants along with strategies to reduce the major risk factors such as obesity and low fruits and vegetable consumption.	[25]
Prevalence of Diabetes among Migrant Women and Duration of Residence in the United Arab Emirates: A Cross Sectional Study	Cross-sectional study among 599 migrants (Filipinos, Arabs and South Asians) women aged 18 years and over at a visa screening center in Al Ain, UAE. South Asians included Indian, Bangladeshis, and Pakistanis.	The prevalence of prediabetes and diabetes among South Asians migrant women were 30.3 and 16.7% respectively.	In adjusted analysis, significant correlates of diabetes included residence in UAE for more than 10 years (OR = 2.74, 95% CI: 1.21–6.20), age 40 years (OR = 3.48, 95% CI: 1.53–7.87) and South Asian nationality (OR 2.10, 95% CI: 0.94–4.70).	Diabetes was highly prevalent among migrant women in the UAE, particularly South Asians. The longer length of residence in UAE is associated with a higher prevalence of diabetes. After ten years of residence, migrant women have three times the prevalence of diabetes compared with more recent arrivals.	There is a lack of validated instruments to measure acculturation amongst migrants in the Gulf region. Future research may aim to develop a contextually and culturally appropriate tool. Further research is required to investigate the dietary and behavioral factors that are contributing to the upward trend in overweight, obesity, and diabetes in migrant women in the UAE. Interventions aimed at the maintenance of a healthy body size and regular assessment of glucose control is recommended.	[26]

Three studies reported HTN prevalence at 31–58% among the South Asian migrants (Indian, Pakistani and Bangladeshi) [23–25]. One study that compared migrants and non-migrants found that migrants were three times as likely to have HTN compared to non-migrants (OR: 3.0, 95% CI: 1.8–4.9) [25]. Migrants were also more likely to be physically inactive (OR: 1.8; 95% CI: 1.2–2.6), and have a family history of HTN (OR: 1.5; 95% CI: 1.1–2.2) [23]. The HTN awareness, treatment, and control were also lower in migrants compared to non-migrants (44% vs. 57% aware, 34% vs. 53% treated and 12% vs. 48% controlled) [25].

Two studies reported the prevalence of DM at 9 and 16.7% [24, 26]. The prevalence of DM was 5%(20/378) among the UAE immigrants who lived there for less than ten years compared to 24%(24/100) among those who lived there for 10 years or more [26]. The longer stay (> 10 years) in the host country (UAE) was associated with a higher odds of DM (OR: 2.7, 95% CI: 1.2–6.2) [26]. High prevalence of overweight (30 and 66%) and obesity (19, and 88%) were reported amongst South Asian male migrants [24–26].

Three of the four studies used “years of residency” as a surrogate measure of acculturation [24, 26]. The studies recommended public health interventions and strategies to improve the awareness and control of HTN and DM among these populations along with regular assessments of blood pressure and/or blood glucose.

### CMD risk factors in home countries

The population with the presence of three or more NCD risk factors (smoking, lack of physical activity, obesity, fruits and vegetables, blood pressure) ranged from 13.5% in Bhutan to 40.0% in Pakistan (Table 2).

**Table 2:** Prevalence of non-communicable diseases risk factors in South Asian Countries

Country (years, references)	Sample	Percentage with three or more risk factors^a^	Mean number of servings consumed on average per day	
			Fruit	Vegetable
Bangladesh (2010) [43]	9275 adults aged 25 years and above	28.3	1.7	2.3
Pakistan (2014–15) [44]	7710 adults aged 18–69 years	40.0	0.6	1.2
Bhutan (2014) [45]	2822 adults aged 18–69 years	13.5	0.7	3.8
Maldives (2011) [46]	1780 adults aged 15–64 years	39.5	1.0	1.0
Nepal (2013) [47]	4143 adults aged 15–69 years	15.1	0.5	1.4
Sri Lanka (2015) [48]	5188 adults aged 18–69 years	18.3	1.3	3.0

^a^Smoking, lack of physical activity, obesity, fruits and vegetables, blood pressure

A systematic review of HTN in South Asia in 2014 reported that about one-third of the population is hypertensive [17]. Fruits and vegetable consumption was remarkably low among people in this region, with the mean number of fruit servings per day ranging from 0.5 in Nepal to 1.7 in Bangladesh, and the mean number of vegetable servings per day ranging from 1.0 in the Maldives to 3.8 in Bhutan. Table 3 summarizes the CMD risk factors in home countries.

**Table 3:** Risk factors for cardio-metabolic diseases in home and host countries

NCD Risk Factors^a^	Sample size	Alcohol use	Tobacco use	Low physical activity	Low fruits and vegetable consumption	Diabetes	Hypertension	Hyperglycemia	Hypercholesterolemia	Overweight/Obesity	Mean BMI	Ref
Home countries												
Nepal (2013)	4143 adults aged 15–69 years	17.4%	18.5%	3.5%	98.9%	3.6%	25.7%	4.1%	22.7%	21.6% overweight and 4.0% obese	22.4	[47]
India (2008)	38,064 adults aged 15- 64 years [49]	5.8 to 14.3% [49]	9.5 to 44.0% [49]	42.3 to 81.2% [49]	75.7 to 98.9% [49]		16.6 to 21.1% [49]	0.6 to 5.9% [49]	27.1% [50]	9.7 to 25.7% overweight, 1.8 to 8.0% obese [49]	20.1 to 22.7 [49]	[49, 50]
Pakistan (2014)	7710 adults aged 18–69 years	–	13.9%	41.5%	96.5%	3.4%	37.0%	–	1.5%	41.3% overweight and 14.9% obese	24.5	[44]
Bangladesh (2010)	9275 adults aged 25 years and above	0.9% (66.7% of this population engaged in binge-drinking)	26.2%	27.0%	95.7%	3.9% self-reported	17.9%	–	–	17.6% overweight	21.5	[43]
Bhutan (2014)	2822 adults aged 18–69 years	42.4%	7.4%	6.4% (51.5% physical inactivity in 2010 a/c to country profile annexed in Oxford report)	66.9%	6.4%	35.7%	10.7%	12.5%	33.0% overweight and 6.2% obese	24	[45]
Afghanistan (2008)	–	–	0.7%	–	–	–	22.5%	–	–	2.2% obese	–	[51]
Sri Lanka (2015)	5188 adults aged 18–69 years	17.9%	15.0%	30.4%	72.5%	7.4%	26.1%	3.8%	23.7%	29.3% overweight and 5.9% obese	22.9	[48]
Maldives (2011)	1780 adults aged 15–64 years	0.9%	18.8%	45.9%	93.6%	4.7%	16.6%	6.2% in 2010 a/c to country profile annexed in Oxford report)	–	37.1% overweight and 11.5% obese	23.7	[46]
Host Countries												
Qatar (2012)	2496 adults aged 18–64 years	–	16.4%	45.9%	91.1%	16.7%	16.4%	5.8%	21.9%	70.1% overweight and 41.4% obese	29.2	[28]
Saudi Arabia (2005)	5000 adults aged 15–64 years	–	24.2% men and 1.4% women	67.7%	91.6% men and 95.3% women	15.8% men and 14.9% women	21.3% total, 24.2% men and 18.5% women	19.6% men and 17.1% women; 18.3% total	18.6% men and 19.7% women (≥5.2 mmol/L)	37.9% men overweight, and 28.3% men obese; 27.6% women overweight, 43.8% women obese	27.0 for men and 29.1 for women	[52]
UAE (2014–2016)		–	28.0% among men and 0.9% among women in 2016 among adults aged 18 years or above [53]	30.2% in 2016 [54]	–	8.0% total, 7.8% men and 8.5% women in 2016 [54]	19.1% total, 21.1% men and 13.3% women in 2014 [55]	–	–	70.6% overweight and 34.5% obese in 2016 4 [54]	–	[53–55]
Jordan (2007)	3654 adults aged 18 years and over	0.9%	29.0%	5.2%	14.2%	16.0%	25.5%	23.8%	36.1%	67.4% overweight,36.5% obese	28.5	[56]
Lebanon (2010)	1982 adults aged 25–64 years	20.5%	38.5%	45.8%	–	11.2%	13.4%	17.9%	71.9%	65.4% overweight or obese	27.5	[27]
Kuwait (2015)	4391 adults aged 18–69 years	0.8%	20.5%	62.6%	83.8%	14.6%	25.1%	6.1%	55.9%	77.2% overweight and 40.2% obese	29.4	[29]
Bahrain (2007)	1769 adults aged 20 to 64 years	–	19.9%	–	–	14.3%	38.2%	12.0%	40.6%	32.9% overweight and 36.3% obese	28.54(6.4)	[57]

^a^Only STEPS surveys results have been used for the table, as this increases comparability with exceptions where data were not available

Definitions of variables used in the table: Alcohol use (consumed alcohol in the past 30 days); Tobacco use (smokes tobacco in any form either daily or occasionally at the time of the survey);Low physical activity (< 600 MET-minutes per week or < 150 min of moderate-intensity activity per week);Low fruits and vegetable consumption (ate less than 5 servings of fruit and/or vegetables on average per day);Diabetes (plasma venous value ≥126 mg/dl or ≥ _7.0 mmol/L or currently on medication for raised blood glucose);Hypertension (SBP ≥ 140 and/or DBP ≥ 90);Hyperglycemia (percentage with raised fasting blood glucose value ≥110 mg/dl and < 126 mg/dl or ≥ 6.1 mmol/L or currently on medication for raised blood glucose);Hypercholesterolemia (percentage with raised total cholesterol ≥5.0 mmol/L or ≥ 190 mg/dl or currently on medication for raised cholesterol);Overweight (≥25 kg/m2 or Obesity ≥30 kg/m2

### CMD risk factors in host countries

The proportion of people having three or more NCD risk factors was 34.1% in Lebanon [27], 50.6% in Qatar [28] and 57.9% in Kuwait [29]. We found a high mean BMI in Qatar (29.2 kg/m^2^), Jordan (28.5 kg/m^2^) and Kuwait (29.4 kg/m^2^). In Qatar, 70% of the population are overweight, and of them 41% are obese. The situation is similar in other Middle Eastern countries, for example, UAE (Overweight 71%, of them obese 35%) and Jordon (Overweight 67%, of them obese 37%). Moreover, the consumption of fruits and vegetables is low. Nearly 91% of Qatari and 84% of Kuwatis had less than the recommended level of fruit and vegetable consumption. The highest DM prevalence was in Qatar (17%) and HTN in Bahrain (38%) (Table 3).

### Migration policies in south Asian countries

Migration is poorly regulated in this region, owing to the lack of robust monitoring infrastructure. None of the South Asian countries have ratified the ILO conventions on migrant workers [15], while only Sri Lanka and Bangladesh have ratified the United Nations (UN) conventions on ‘*The International Convention on the Protection of the Rights of All Migrant Workers and Members of Their Families’* (2003) [30]. The ILO and the UN conventions are international legal instruments for migrants, and the stated rights can be claimed only if the home country has ratified them [15]. The World Health Organization (WHO) and the IOM called for monitoring of chronic diseases among migrants in 2010 [3]. UN post-2015 Development Agenda highlighted that maintaining health during migration is crucial to achieving a right to health for migrants. However, these agendas have not been substantiated.

The policy documents (periodic plans and policies) from eight South Asian countries pertaining to CMDs are summarized in Table 4 (see Additional file 2 for reviewed literature). The national policies of Nepal, Sri Lanka and Bangladesh states provision for pre-departure medical check-u, but details are lacking. See the Additional file 3 for the summary of migration policies in the South Asian region). Afghanistan, Pakistan, and Maldives do not have specific policies to address cardio-metabolic diseases for migrant workers. Bhutan has a policy of “medical fitness certificate” for inbound migrants working in Bhutan but not for the Bhutanese migrant workers seeking employment abroad. Similarly, “India’s national policy on safety, health, and environment at workplace (2009)” underscores the importance of addressing migrant health and there is no specific policy or strategy for screening, prevention or treatments of NCDs.

**Table 4:** Recommendation for the host and home country governments across different time-frames

Time-frame	Issues	Actors		Recommendations
		HOME	HOST	
Short term	Heat and exhaustion	+		Providing adequate hydration at work
	Heat and exhaustion		+	Providing heat shields
	False reports	+		Addressing issues regarding false medical reports by enforcing monitoring on screening centers
Intermediate	Low awareness	+	+	Coordination with provincial and district health offices to raise awareness on importance of lifestyle changes, physical activity and medical checkups even before the migration cycle starts
	Low awareness			Coordination with companies, recruiting agencies and local health offices to raise awareness on lifestyle, physical activity and medical check ups
	Surveillance and monitoring	+	+	Tracking the out-bound and in-bound migrants and addressing their health outcomes using routine health registers
	Surveillance and monitoring			The information obtained from health assessment should be shared not just within migration authorities but also across health sector, and integrated within the health system in host and home country.
	Adherence to medication and treatment	+	+	For those with existing CMD, counselling on adherence to medication, lifestyle changes and physical activity
Long term	Limited health promoting facilities	+	+	Health and wellbeing centers targeting outgoing and in-coming migrants
	Limited insurance coverage	+	+	The insurance package should cover the health expenses when returning home with CMD, and coverage for any disability/deaths.
	Low political priority		+	Cooperation at the ministerial level to accord migrant’s cardio metabolic health as a top priority. The first step will on providing exercise facility, adequate space to live and provision for adequate nutrition and hydration at work.

*Abbreviation*: *CMD* cardio-metabolic disease

+ shows where the actions are needed

Currently, only a few diseases are screened prior to migration, the majority of which are communicable diseases (e.g. TB, STIs). In addition, the workers are not followed up and their continued health care is neglected at work. The pre-departure medical check-up depends upon the requirement of the host countries [31]. For example, migrants to the Gulf countries are tested for the following conditions in the home countries and again in the host countries [31]: HIV, sexually transmitted infections (STIs), tuberculosis, bronchial asthma, peptic ulcer disease, malaria, leprosy, cancer, epilepsy, hearing problem, hepatitis, and psychiatric illness and pregnancy with limited focus on heart disease, DM, HTN and kidney disease. The Gulf Cooperation Council has adopted a law of “Pre-departure Medical Check Up for the incoming migrant workers in the Gulf States”, which is implemented through the Gulf Approved Medical Centers Association (GAMCA) [31].

An in-depth analysis for Nepal concerning migrants’ health is shown in panel 1 (see Additional file 4 for reviewed literature). The case of Nepal could be important, given the relatively younger migrant population and higher per capita contribution to Nepal’s Gross Domestic Product (GDP) (remittances contributed to 25% of total GDP in 2013) [32]. Furthermore, Nepal is one of the two low-income countries in this region, with the second lowest per capita GDP (US$ 730) after Afghanistan [33]. India and Bangladesh have recently graduated from low-income country status to lower-middle income countries.

#### Panel 1: migration policies in Nepal

None of the 18 documents and two web pages that we reviewed explicitly advocated or mandated monitoring for CMD. The National Health Policy of Nepal, 2014 states health as a human right for Nepalese in general, and does not speak specifically to migrant population. The “Multi-Sectoral Action Plan” for the prevention and control of NCDs (2014–2020) mandates the screening of NCDs in Nepali migrant workers; and providing counseling and clinical services as required. However, it is not specified which NCDs are included in the policy. Nepal Health Sector Programme strategy II (2010–2015) addresses unmet family planning needs, and prevention of sexual transmission of HIV among male labor migrants and their partners; but has no mention of NCDs.

Nepal’s National Youth Policy 2015 also acknowledges the health of the youth in general and remains silent on NCDs and health of young migrants. Foreign Employment Act, 2007 and 2011, mandates pre-departure health certification from a health institution approved by the Government of Nepal without specifying the conditions to be screened. Nepal’s foreign affairs (2015–2016) are primarily focused on increasing the bilateral relations with employment destination countries and making foreign employment less burdensome for the workers. It remains silent on any bilateral policies to address health needs of these workers. Recently, the National Occupational Safety and Health Policy 2016 has been proposed, which focuses on promoting safety and health of labor employed in Nepal, however, does not encompass the health and safety of migrant workers.

Nepal Labor Force Survey 2008, Nepal living standards survey 2011, and Nepal Demographic and Health Survey, 2006 and 2011, reported migration from demographic and socioeconomic aspects but not from health. The “Labour migration for employment a status report for Nepal” for the year 2013/2014 and 2014/2015″ provide a comprehensive status of Nepalese migration along with a summary of various government-led initiatives at the policy and structural levels to promote safe migration. But, there is a lack of policies on migrant’s health.

The IOM, in collaboration with the Ministry of Health of Nepal, operates the Migration Health Department (MHD) in two cities of Nepal, viz. Damak and Kathmandu [34]. The MHD provides assistance in medical screening, counseling, health education, and preparation of immigration medical documents. However, MHD’s primary target population is refugees, not migrant workers. It screens diseases at the destination country, which are primarily infectious diseases. Likewise, ILO in Nepal promotes safe and healthy conditions in the workplace within Nepal by facilitating application of the International Labour Standards; supporting to formulate national labor legislation; and promoting social dialogue, social justice, and decent work environment.

## Discussion

There is a high burden of CMDs including HTN and Diabetes among the Middle East immigrants from South Asia, coinciding with similar burden in the home and host country. There is a lack of attention on migrant’s cardio-metabolic health in the policies and programs by both home and host country governments. This is also reflected in the in-depth analysis of policy documents from Nepal—a country where migrant workforce contribute up to a quarter of GDP.

### Future risk of CMD in migrants

CMD is the leading cause of mortality, morbidity, and disability in South Asia [35]. In 2008, it was the leading cause of death in India and Bangladesh and accounted for 34 and 53% of all deaths in Maldives and Bhutan, respectively [36]. Migrants shared similar risk factors with the home and host country populations. Therefore, those migrants to the Middle East without preexisting CMD may develop it, and those with pre-existing CMD may worsen their condition.

Five thousand Nepalese migrants died working abroad between 2008 and 2014 [37], approximately 29% of these deaths were due to cardiac arrest or heart attack [38], highlighting the immense burden of CMDs and their risk factors in this population [38, 39]. However, an earlier report showed a non-difference in death rates between Nepalese migrant workers and general population aged 15–34 years at home [38]. Given that migrants are comparatively healthier, and physically fit for work, the same death rate among migrants still means a higher than expected death rate had they not have migrated. In short-term, migrant’s modest salary would give better economic leverage at work and home. However, in the long-term it has an adverse impact on migrants’ health, especially among those with preexisting health conditions. ‘Sedentarism-corporates complex’ [40] of a little physical activity coupled with unhealthy eating and stress increases cardiovascular disease risk in this population. Furthermore, consumption of unhealthy amounts of alcohol [4, 41] as a coping mechanism to reduce stress and entertainment away from their home is well recognized problem. When the migrants come back home, they also bring the unhealthy practices that can influence health behaviors in their society.

Further, the potential impact of heat stress and climate change on the CMD risk among migrants in the Middle East is less studied so far. Heat stress can deter physical health among those with existing CMDs [42] in the Middle East where the daily average temperature exceeds what migrants normally experience at home. Therefore, promoting safe working environment seems important to reduce the disease burden as well as prevent avoidable deaths. A continuum of intervention is necessary to address the transport of CMD from work to home, and home to work.

### Accessing health services for migrants

The pre-departure medical screenings are aimed to assess the worker’s fitness to work rather than to promote their overall wellbeing. Furthermore, there is no policy on migrant health in the host country that would protect the rights of migrants to prevention, treatment, and care. South Asia has also been criticized to have intentionally neglected the migrants’ rights for fear of losing labour markets in the host countries [8]. Even if there are rules that mandate all migrant workers to receive certification of medical approval, migrants may often provide false reports due to a dysfunctional monitoring system. Furthermore, the economic demand of the migrants in the host country trumps the priority over spending the little available money for healthcare. There is also a lack of awareness and counseling on a healthy lifestyle in the home country.

Only a third of Nepali migrants in the Middle East had health insurance [16]. In a study among Sri Lankan female migrant domestic workers, the majority of the workers were not aware of entitlements under the insurance scheme [8]. Only few workers asked for compensation after injuries or illnesses. Those who were working under the second contract had less favourable employment terms compared to those working under their first contract [8]. Furthermore, access to medical treatment was at the employer’s discretion although stipulated in the employment contract. Only limited women were entitled insurance benefits, though it was mandatory in the paper. The employers violated many of the provisions in the contracts such as the duration of working hours, resting times, food, and humane treatment at work [8]. Employees feared that reporting poor health conditions (to their employers) will lead to employment termination and repatriation [16].

The governments and health providers at host countries are not prepared to handle a large number of arrivals. The migrant’s health care access is further limited by stricter laws for commuting, language and cultural barriers, and lack of access to prepaid health services. For example, a hotline to report violations or refusal of care in Lebanon operated only in the Arabic language whereas many migrants Arabic non-speakers [8]. Media have constantly reported the negative perception of the host population on migrants utilizing services in the host countries [3], that can increase the risk of violence to migrants.

## Way forwards

A stronger commitment to the health of migrants from both the home and host countries level could be groundbreaking in migrants’ health. This is beneficial to both the migrants and the host countries because of contribution to the increased productivity from a healthier, happier and a vibrant migrant workforce. Furthermore, the host government should provide workplace health promotion, on arrival health screening and long-term healthcare programs to the migrants. Additionally, the governments should formulate policies to prevent and manage cardio-metabolic health for migrants, as well as increase funding for programs and research in migrants’ health. These are further discussed in Table 4, where we have identified several issues that can be addressed in short, intermediate and long term. For example, the counseling to outbound and inbound migrants can be a cost-effective solution to address the low awareness and utilization of screening services in the short term learning from similar initiatives conducted in communicable disease context [3]. However, cautions need to be taken to avoid the use of screening as a tool to disqualify migrants from their current work, and country of current residence.

**Fig. 2:**
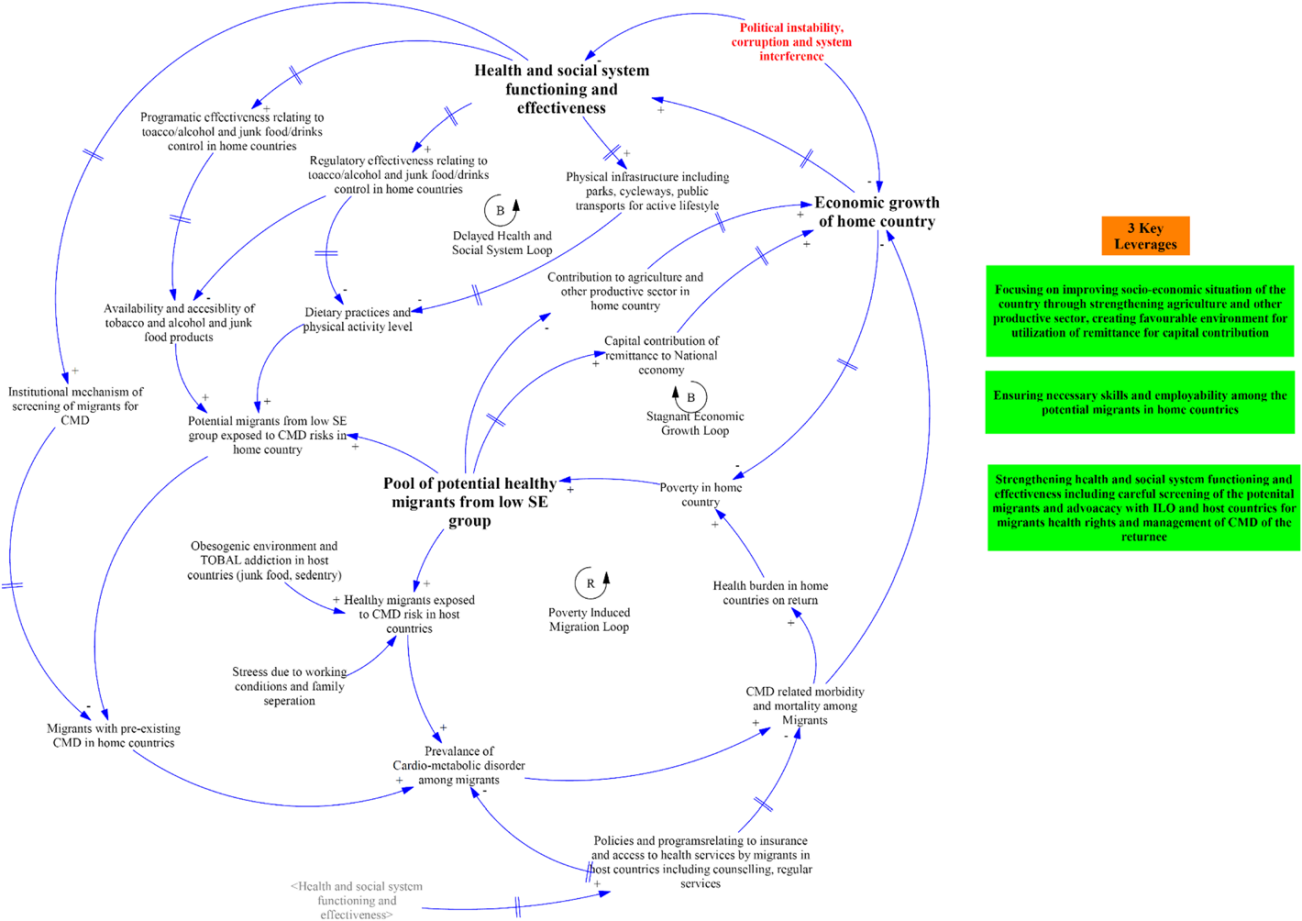
System map of social determinants of CMD among labour migrants of South Asia Region. ‘R’ denotes a reinforcing loop and ‘B’ denotes a balancing loop. More description about them is available in reference [12] and [13]. *Abbreviations: CMD: cardio metabolic disease;’* SE: socio-economic

Figure 2 demonstrates the system map to summarize the key social determinants of CMD among labour migrants and identify the leverages for systemic actions. There are overall three loops, i) delayed health and social system loop, and ii) stagnant economic growth loop, and iii) poverty induced migration loop. Each loop summarizes the factors that reinforce or balances each other in a given policy context. For example, the first loop shows the effect of health and social system on migrant’s lifestyle and their overall health. The second loop shows the vicious cycle of poor cardiometabolic health and poverty, and the third shows the link between the migrant’s health and overall economic growth of the home country. A total of three leverages are identified; first, improving socio-economic wellbeing through reforms on agriculture, labour, and environmental sectors; second, imparting skills and increasing employability among the migrants in home countries; and third, recommends strengthening of health system functioning including the screening of potential migrants in collaboration with concerned stakeholders.

## Limitations

Our review has some limitations. First, our Medline search found only four original research articles that reported CMD among labour migrants of South Asian origin in the Middle Eastern countries. Therefore, we did not pool the CMD risk factors and their trends over time. Second, we used only STEPS survey reports and WHO country profiles for summarizing the burden of risk factors in the home and the host country populations; and did not include other small intra-country studies. And the third, the quality of the included studies was not assessed due to the limited number of studies.

## Conclusions

We found limited literature on the burden of CMD among the South Asian migrant population in the Middle East. Risk factors of CMD among migrant populations varied according to the country. We found that HTN was the most reported condition among the migrants followed by DM. This coincides with a high burden of hypertension and diabetes among populations in both home and host countries. We did not find all-cause and cause specific diseases or mortality rates among migrants during our review of the literature, suggesting that there is a need for prospective studies in this area. We found a limited focus on migrant’s health beyond the communicable disease from the policy analysis. The growing burden of CMDs in migrant represents a serious public health challenge for many South Asian countries. Migrant people had poor access to health care in the host country, including lack of preparedness of government and health service providers in dealing with a large number of arrivals. Awareness on migrants’ CMD risk, targeted screening, immunization record checks, and treatment from home to host country, and during their return from host to home country should start immediately by the contracting companies and governments. Stricter pre-medical examination, follow up visits and counseling should be implemented by the governments. Further, there is a need for advocacy and lobbying (to health ministries in the Middle East) for better placement and better working environment by leveraging organizations committed to monitoring international labor standards. The research focused on migrants’ health with an emphasis on CMD is urgently required to formulate evidence-based policies and to curb the escalating burden of CMDs.

## Additional files

Additional file 1: Terms used for searches in Pubmed/Medline are listed here. (DOCX 19 kb)

Additional file 2: Documents reviewed for policy analysis for countries in South Asia are listed here. (DOCX 19 kb)

Additional file 3: Summary of policy analysis in South Asia region are listed here. (DOCX 17 kb)

Additional file 4: Documents reviewed for panel 1 are listed here. (DOCX 17 kb)
