# Supplementary material for: Cardio-metabolic disease risk factors among South Asian labour migrants to the Middle East: a scoping review and policy analysis
Source: Global Health. 2019 May 2;15:33. doi: 10.1186/s12992-019-0468-8 (PMC6498694; doi:10.1186/s12992-019-0468-8)
Supplement: Supplementary file 1 — Terms used for searches in Pubmed/Medline are listed here. (DOCX 19 kb) [file 12992_2019_468_MOESM1_ESM.docx]

**Additional file 1: : Terms used for searches in Pubmed/Medline are listed here.**

"Transients and Migrants"[Mesh] AND "Cardiovascular Diseases"[Mesh]) AND "Middle East"[Mesh] 15 results

("Transients and Migrants"[Mesh] AND "Cardiovascular Diseases"[Mesh]) AND "Asia, Western"[Mesh] 27 results

"Diabetes Mellitus"[Mesh] AND "Transients and Migrants"[Mesh] AND "Middle East"[Mesh] 6 results

"Diabetes Mellitus"[Mesh] AND "Transients and Migrants"[Mesh] AND "Asia, Western"[Mesh] 14 results
