# Supplementary material for: Cardio-metabolic disease risk factors among South Asian labour migrants to the Middle East: a scoping review and policy analysis
Source: Global Health. 2019 May 2;15:33. doi: 10.1186/s12992-019-0468-8 (PMC6498694; doi:10.1186/s12992-019-0468-8)
Supplement: Supplementary file 2 — Documents reviewed for policy analysis for countries in South Asia are listed here. (DOCX 19 kb) [file 12992_2019_468_MOESM2_ESM.docx]

**Additional file 2: Documents reviewed for policy analysis in South Asia are listed here.**

| **Countries** | **List of policies reviewed for each country** | **Reference** |
| --- | --- | --- |
| Nepal | Multi-sectoral Action Plan for The Prevention and Control of NCDs (2014-2020) | [1] |
|  | National Health Policy 1991 | [2] |
|  | National Health Policy 2014 | [3] |
|  | Nepal’s Foreign Employment Act 1985 | [4] |
|  | Nepal’s Foreign Employment Act, 2007 | [5] |
|  | Nepal’s Foreign Employment Policy 2012 | [6] |
|  | Labour and Employment Policy, 2005 | [7] |
| Bangladesh | Strategic Plan for Surveillance and Prevention of Non-Communicable Diseases in Bangladesh 2011-2015 | [8] |
|  | National Health Policy 2008 | [9] |
|  | National Health Policy 2011 | [10] |
|  | The Emigration Ordinance, 1982 | [11] |
|  | Overseas Employment and Migrants Act 2013 | [12] |
|  | National Labour Policy 2010 | [13] |
| Pakistan | National Action Plan for Prevention and Control of Non-Communicable Diseases and Health Promotion in Pakistan, 2004 | [14] |
|  | National Health Policy 2009 | [15] |
|  | Labour Protection Policy 2006 | [16] |
|  | Labour Policy 2010 | [17] |
| Bhutan | National Health Policy 2011 | [18] |
|  | Immigration Rules & Regulations of The Kingdom of Bhutan 2015 | [19] |
|  | The Immigration Act of The Kingdom of Bhutan, 2007 | [20] |
|  | Labour and Employment Act of Bhutan 2007 | [21] |
|  | National Employment Policy 2013 | [22] |
|  | Handbook on Recruitment and Employment of Foreign Workers | [23] |
| Maldives | Multi-Sectoral Action Plan for The Prevention and Control of Non-Communicable Diseases in Maldives 2016-2020 | [24] |
|  | Maldives Health Master Plan 2016- 2025 | [25] |
|  | The Maldives Immigration Act, 2007 | [26] |
|  | Employment Act, 2008 | [27] |
|  | Fifth Amendment to The Employment Act, 2016 | [28] |
| Sri Lanka | The National Policy & Strategic Framework for Prevention and Control of Chronic Non-Communicable Diseases 2009 | [29] |
|  | National Health Policy 2016-2025. | [30] |
|  | National Labour Migration Policy for Sri Lanka, 2008 | [31] |
|  | Sri Lanka National Migration Health Policy, 2012 | [32] |
| Afghanistan | National Strategy for Prevention and Control of Non-communicable diseases (NCDs) 2013-2018 | [33] |
|  | National Strategy for Prevention and Control of Non-communicable diseases (NCDs) 2015-2020 | [34] |
|  | National Health Policy 2015 - 2020 | [35] |
|  | National Health Strategy 2016‒2020 | [36] |
|  | Afghanistan Migration Profile 2014 | [37] |
|  | Labor Law 2007 | [38] |
|  | National Labour Policy 2012 | [39] |
| India | National Action Plan for Prevention and Control of NCDs in India 2013-2020 | [40] |
|  | National Health Policy, 2017 | [41] |
|  | The Emigration Act, 1983 | [42] |
|  | National Policy on Safety, Health and Environment at Work Place, 2009 | [43] |

**References**

1. Multisectoral Action Plan for the Prevention and Control of Non Communicable Diseases (2014-2020). Kathmandu, Nepal: Government of Nepal; World Health Organization Country Office for Nepal; 2014.
2. National Health Policy, 2048 (1991). Kathmandu, Nepal: Ministry of Health and population, Government of Nepal; 1991.
3. National Health Policy, 2071 (2014). Kathmandu, Nepal: Ministry of Health and population, Government of Nepal; 2014.
4. Foreign Employment Act, 2042 (1985). Kathmandu, Nepal: Ministry of Labour and Employment, Government of Nepal; 1985.
5. Foreign Employment Act, 2064 (2007). Kathmandu, Nepal: Ministry of Labour and Employment, Government of Nepal; 2007.
6. Foreign Employment Policy 2068. Kathmandu, Nepal: Ministry of Labour and Employment, Government of Nepal; 2012.
7. Labour and Employment Policy, 2062 (2005). Kathmandu, Nepal: Ministry of Labour and Employment, Government of Nepal; 2005.
8. Strategic Plan for Surveillance and Prevention of Non Communicable Diseases in Bangladesh 2011-2015. Dhaka, Bangladesh WHO/SEARO/Country Office for Bangladesh and Ministry of Health & Family Welfare, Bangladesh; 2011.
9. National Health Policy 2008. Dhaka, Bangladesh: Ministry of Health and Family Welfare, Government of the People’s Republic of Bangladesh; 2008.
10. National Health Policy 2011. Dhaka, Bangladesh: Ministry of Health and Family Welfare, Government of the People’s Republic of Bangladesh; 2011.
11. Bangladesh: The Emigration Ordinance, 1982. Dhaka, Bangladesh: National Legislative Bodies / National Authorities; 1982.
12. Overseas Employment and Migrants Act 2013 *(Act No. VLVIII of 2013)*. Dhaka, Bangladesh: Legislative and Parliamentary Affairs Division, Bangladesh; 2013.
13. National Labour Policy 2010. Dhaka, Bangladesh: Government of the People’s Republic of Bangladesh; 2010.
14. National Action Plan for Prevention and Control of Non-Communicable Diseases and Health Promotion in Pakistan. Islamabad, Pakistan: Ministry of Health, Government of Pakistan; WHO, Pakistan office, and Heartfile; 2004.
15. National Health Policy 2009. Islamabad, Pakistan: Ministry of Health, Government of Pakistan; 2009.
16. Labour Protection Policy 2006. Islamabad, Pakistan: Ministry of Labour, Manpower and Overseas Pakistanis, Government of Pakistan; 2006.
17. Pakistan: Labour Policy 2010. Islamabad, Pakistan: Government of Pakistan; 2010.
18. National Health Policy, 2011 - Bhutan. Thimphu, Bhutan: Ministry of Health, Royal Government of Bhutan; 2011.
19. Immigration Rules & Regulations of The Kingdom Of Bhutan. Thimphu, Bhutan: Ministry of Foreign Affairs, Royal Government of Bhutan; 2015.
20. The Immigration Act of The Kingdom of Bhutan, 2007. Thimphu, Bhutan: Royal Government of Bhutan; 2007.
21. Labour and Employment Act of Bhutan 2007. Thimphu, Bhutan: Royal Government of Bhutan; 2007.
22. National Employment Policy 2013. Thimphu, Bhutan: Ministry of Labour and Human Resources, Royal Government of Bhutan; 2013.
23. Handbook on Recruitment and Employment of Foreign Workers. Thimphu, Bhutan: Department of Labour, Ministry of Labour and Human Resources, Royal Government of Bhutan.
24. Multi-sectoral Action Plan for the Prevention and Control of Noncommunicable Diseases in Maldives (2016-2020). Male, Maldives: Ministry of Health; Republic of Maldives; 2015.
25. Maldives Health Master Plan 2016- 2025. Male, Maldives: Ministry of Health; Republic of Maldives; 2014.
26. The Maldives Immigration Act, 2007 (*Act No 1/2007)*. Male, Maldives: Ministry of Economic Development, Republic of Maldives; 2007.
27. Employment Act, 2008 (Act No. 2/2008). Male, Maldives: Ministry of Human Resources Youth and Sports, Maldives; 2008.
28. Fifth Amendment to the Employment Act, 2016. Male, Maldives: Government Gazette, Ministry of Human Resources Youth and Sports, Maldives; 2016.
29. The National Policy & Strategic Framework for Prevention and Control of Chronic Non-Communicable Diseases 2009. Colombo, Sri Lanka: Ministry of Healthcare and Nutrition, Sri Lanka; 2009.
30. National Health Policy 2016-2025. Colombo, Sri Lanka: Ministry of Healthcare and Nutrition, Sri Lanka; 2016.
31. National Labour Migration Policy for Sri Lanka, 2008. Colombo, Sri Lanka: Ministry for Foreign Employment Promotion And Welfare; 2008.
32. Sri Lanka National Migration Health Policy. Colombo, Sri Lanka: Ministry of Healthcare and Nutrition, Sri Lanka; International Organization on Migration (IOM); 2012.
33. National Strategy for Prevention and Control of Noncommunicable Diseases (NCDs) 2013-2018. Kabul, Afghanistan: Noncommunicable Diseases Prevention and Control Department, General Directorate of Preventive Medicine, Ministry of Public Health Afghanistan; 2013.
34. National Strategy for Prevention and Control of Noncommunicable Diseases (NCDs) 2015-2020. Kabul, Afghanistan: Noncommunicable Diseases Prevention and Control Department, General Directorate of Preventive Medicine, Ministry of Public Health Afghanistan; 2015.
35. Afghanistan National Health Policy 2015-2020. Kabul, Afghanistan: Ministry of Public Health, Islamic Republic of Afghanistan; 2015.
36. National Health Strategy 2016‒2020. Kabul, Afghanistan: Ministry of Public Health, Islamic Republic of Afghanistan; 2016.
37. Marchand K, Siegel M, Kuschminder K, Majidi N, Vanore M, Buil C: Afghanistan Migration Profile. Kabul, Afghanistan: International Organization for Migration Afghanistan; 2014.
38. Labour Law (No. 35 of 2007). Kabul, Afghanistan: Ministry of Justice, Islamic Republic of Afghanistan; 2007.
39. National Labour Policy. Kabul, Afghanistan: Ministry of Labor, Social Affairs, Martyrs & Disabled, Government of Islamic Republic of Afghanistan; 2012.
40. National Action Plan and Monitoring Framework for Prevention and Control of Noncommunicable Diseases (NCDs) in India. New Delhi, India: Ministry of Health and Family Welfare, Government of India; 2013.
41. National Health Policy, 2017. New Delhi, India: Ministry of Health & Family Welfare, Government of India; 2017.
42. Emigration Act, 1983. New Delhi, India: National Legislative Bodies / National Authorities, Government of India; 1983.
43. National Policy on Safety, Health and Environment at Workplace. New Delhi, India: Ministry of Labour and Employment, Government of India; 2009.
