# Supplementary material for: Cardio-metabolic disease risk factors among South Asian labour migrants to the Middle East: a scoping review and policy analysis
Source: Global Health. 2019 May 2;15:33. doi: 10.1186/s12992-019-0468-8 (PMC6498694; doi:10.1186/s12992-019-0468-8)
Supplement: Supplementary file 3 — Summary of policy analysis in South Asia region are listed here. (DOCX 17 kb) [file 12992_2019_468_MOESM3_ESM.docx]

**Additional file 3: Summary of migrant related policies from South Asia region are shown here.**

| Countries | HTN | MD | CVD | Documents reviewed(n) | Policies excerpts |
| --- | --- | --- | --- | --- | --- |
| Nepal | NCD+ | NCD+ | NCD+ | 7 | Foreign Employment Act, 2007 and 2011, mandates pre-departure health certification of workers. Multi-sectoral action `plan for the prevention and control of NCDs (2014-2020) also advocates for NCDs screening for Nepali migrant workers and to provide counseling and clinical services as required. Since NCD is a very diverse group, it is not clear which conditions are specifically screened. |
| Bangladesh | NCD+ | NCD+ | NCD+ | 6 | Bangladesh’s Expatriates Welfare and Overseas Employment Policy (2016) adopted the “Health Checkup Policy for potential Bangladeshi Migrant Workers”. But there is no specification on the diseases screened. |
| Pakistan | x | x | x | 4 | Pakistan does not have any specific policy related to HTN, DM or CVD for migrant workers declared in the reviewed policies. |
| Bhutan | x | x | x | 6 | Bhutan has a policy of “medical fitness certificate” for foreign workers seeking employment in Bhutan, which has been defined in “the immigration act of the kingdom of Bhutan (2007)” and “immigration rules & regulations of the kingdom of Bhutan (2015)”. However, Bhutan does not have any specific policies for Bhutanese migrant workers seeking foreign employment. |
| Maldives | x | x | x | 5 | Maldives does not have any specific policy related to HTN, DM or CVD for migrant workers declared in the reviewed policies. |
| Sri Lanka | NCD+ | NCD+ | NCD+ | 4 | Among the countries reviewed, Sri Lanka is the only country to have National Migration Health Policy (2012), which aims to ensure the health of migrants throughout the migration cycle. Some of the key strategy includes: develop and implement a comprehensive and standardized health assessment for out bound migrant populations of Sri Lankan origin at the pre-departure stage and provide continuity of care; voluntary health assessments for returnee migrants; implement a system for monitoring, assessment and surveillance of all in bound migrants prior to arrival or soon after arrival in the country to address diseases of public health concern to Sri Lanka. Again, the policy is not specific to any NCDs or those of our interest. |
| Afghanistan | x | x | x | 7 | Afghanistan doesn’t have any specific policy related to HTN, DM or CVD for migrant workers declared in the reviewed policies. |
| India | x | x | x | 4 | Although India’s “national policy on safety, health and environment at work place (2009)” realizes migrant workers to work in risk prone conditions there are no specific policy or strategy for screening, prevention or treatments of NCDs among these population. |

Notes: x indicates that the country doesn’t have any policy related to Hypertension (HTN), Diabetes Mellitus (DM) or Cardiovascular Diseases (CVD) for migrant workers. NCD+ indicates that the country has a policy mandating pre-departure health certification of migrant workers but without any specifications of the conditions screened for the certification. Abbreviation: NCDs, non-communicable disease
