# Supplementary material for: Cardio-metabolic disease risk factors among South Asian labour migrants to the Middle East: a scoping review and policy analysis
Source: Global Health. 2019 May 2;15:33. doi: 10.1186/s12992-019-0468-8 (PMC6498694; doi:10.1186/s12992-019-0468-8)
Supplement: Supplementary file 4 — Documents reviewed for panel 1 are listed here. (DOCX 17 kb) [file 12992_2019_468_MOESM4_ESM.docx]

**Additional file 4: Documents reviewed for policy analysis on Nepal are listed here.**

| **Documents** | **Reference** |
| --- | --- |
| Nepal’s National Health Policy 2048 (1991) | [1] |
| Nepal’s National Health Policy 2071 (2014) | [2] |
| Nepal Health Sector Programme Implementation Plan 2004 – 2009 | [3] |
| Nepal Health Sector Programme 2 (NHSP II) 2010 – 2015 | [4] |
| Nepal Health Sector Strategy 2015-20 | [5] |
| Multi-sectoral action plan for the prevention and control of NCDs (2014-2020) | [6] |
| National Youth Policy 2072 (2015) | [7] |
| Foreign Employment Act 2042 (1985) | [8] |
| Foreign Employment Act 2064 (2007) | [9] |
| Foreign Employment Policy 2068 (2012) | [10] |
| Nepal’s foreign affairs (2015-2016) | [11] |
| Nepal labour force survey 2008 | [12] |
| Nepal living standards survey 2010/11 | [13] |
| Nepal Demographic and Health Survey, 2006 | [14] |
| Nepal Demographic and Health Survey, 2011 | [15] |
| Labor migration for employment a status report for Nepal: 2013/2014 | [16] |
| Labor migration for employment a status report for Nepal: 2014/2015 | [17] |
| National Occupational Safety and Health Policy (2073) | [18] |
| Website of International Organization for Migration (IOM) | <http://www.nepal.iom.int/> |
| Website of International Labor Organization (ILO) | <http://www.ilo.org/kathmandu/lang--en/index.htm> |

**References**

1. National Health Policy, 2048 (1991). Kathmandu, Nepal: Ministry of Health and population, Government of Nepal; 1991.
2. National Health Policy, 2071 (2014). Kathmandu, Nepal: Ministry of Health and population, Government of Nepal; 2014.
3. Nepal Health Sector Program – Implementation Plan (NHSP-IP) 2004 – 2009. Kathmandu, Nepal: Ministry of Health, Government of Nepal; 2004.
4. Nepal Health Sector Program – II (NHSP -II) 2010 – 2015. Kathmandu, Nepal: Ministry of Health, Government of Nepal; 2010.
5. Nepal Health Sector Strategy 2015-20. Kathmandu, Nepal: Ministry of Health, Government of Nepal; 2015.
6. Multisectoral Action Plan for the Prevention and Control of Non Communicable Diseases (2014-2020). Kathmandu, Nepal: Government of Nepal; World Health Organization Country Office for Nepal; 2014.
7. National Youth Policy, 2072 (2015). Kathmandu, Nepal: Nepal Government, Ministry of Youth and Sports; 2015.
8. Foreign Employment Act, 2042 (1985). Kathmandu, Nepal: Ministry of Labour and Employment, Government of Nepal; 1985.
9. Foreign Employment Act, 2064 (2007). Kathmandu, Nepal: Ministry of Labour and Employment, Government of Nepal; 2007.
10. Foreign Employment Policy 2068. Kathmandu, Nepal: Ministry of Labour and Employment, Government of Nepal; 2012.
11. Report on Nepal's Foreign Affairs (2015-2016). Kathmandu, Nepal: Ministry of Foreign Affairs, Government of Nepal; 2016.
12. Nepal Labour Force Survey 2008. Kathmandu, Nepal: Central Bureau of Statistics, National Planning Commission Secretariat, Government of Nepal; 2009.
13. Nepal Living Standards Survey 2010/11. Kathmandu, Nepal: Central Bureau of Statistics, National Planning Commission Secretariat, Government of Nepal; 2011.
14. Nepal Demographic and Health Survey 2006. Kathmandu, Nepal: Ministry of Health and Population, Government of Nepal; New ERA; Macro International Inc.; 2007.
15. Nepal Demographic and Health Survey 2011. Kathmandu, Nepal: Ministry of Health and Population, Government of Nepal; New ERA; Macro International Inc.; 2012.
16. Labour Migration for Employment: A Status Report for Nepal: 2013/2014. Kathmandu, Nepal: Department of Foreign Employment, Ministry of Labour and Employment, Government of Nepal; 2014.
17. Labour Migration for Employment: A Status Report for Nepal: 2014/2015. Kathmandu, Nepal: Department of Foreign Employment, Ministry of Labour and Employment, Government of Nepal; 2016.
18. National Occupational Safety and Health Policy. Kathmandu, Nepal: Ministry of Labour and Employment, Government of Nepal; 2016
